# Double Q‐Learning for Intelligent Multi‐Drug Scheduling in Cancer Chemotherapy Optimisation

**DOI:** 10.1049/syb2.70083

**Published:** 2026-08-03

**Authors:** Behnoush Alizade, Ahmad Hajipour

**Affiliations:** ^1^ Faculty of Electrical & Computer Engineering Hakim Sabzevari University Sabzevar Iran

**Keywords:** learning (artificial intelligence), medical control systems, nonlinear dynamical systems, optimal control, tumours

## Abstract

Chemotherapy scheduling poses a challenging control problem due to the need to suppress tumour growth whilst maintaining systemic toxicity within clinically acceptable limits. This study develops a double Q‐learning–based controller for optimising daily dosing of a three‐drug regimen consisting of cisplatin, docetaxel and irinotecan. A pharmacokinetics–pharmacodynamics (PK/PD) tumour model with eight resistance states is used as the simulation environment. The controller aims to minimise tumour burden whilst enforcing strict toxicity constraints aligned with clinical dosing guidelines. Simulation results show that double Q‐learning substantially outperforms classical Q‐learning, achieving near‐complete tumour suppression within the simulation framework, corresponding to a residual tumour fraction on the order of $10^{-6}$ (approximately six orders of magnitude reduction) whilst maintaining toxicity within predefined constraints. Robustness analyses under physiological parameter variations of up to ±50% and under abrupt disturbance events further demonstrate that the double Q‐learning policy preserves stable closed‐loop behaviour within the simulation environment and exhibits strong resilience to uncertainty. Overall, the results indicate that double Q‐learning provides a proof‐of‐concept framework for adaptive chemotherapy optimisation, with potential for future investigation in reinforcement learning‐based chemotherapy optimisation frameworks.

## Graphical abstract

A double Q‐learning framework is proposed for optimal multidrug chemotherapy scheduling using a closed‐loop pharmacokinetic–pharmacodynamic (PK/PD) model. The agent learns personalised dose combinations of cisplatin, docetaxel and irinotecan to maximise tumour suppression whilst minimising treatment toxicity. Simulation results demonstrate highly effective tumour reduction with robust performance under uncertainty.

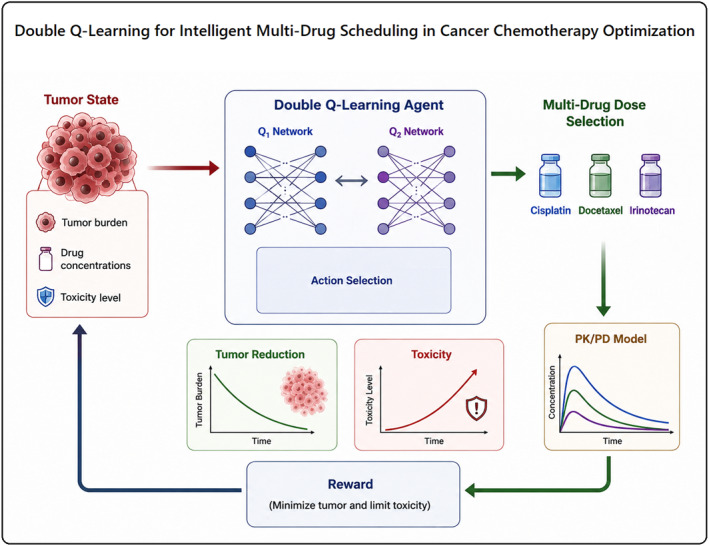

## 1 Introduction

Cancer remains a major global health challenge despite notable advancements in diagnostic and therapeutic modalities over recent decades. It is currently responsible for approximately one out of every sixth deaths worldwide, and global incidence continues to rise [1, 2, 3]. Although therapeutic approaches have expanded to include immunotherapy, radiotherapy, targeted agents and combination treatments [4], chemotherapy remains a cornerstone for managing advanced or recurrent malignancies, including non‐small‐cell lung cancer (NSCLC).

Mathematical modelling has played an important role in understanding tumour evolution and treatment response. Earlier models relied on homogeneous growth functions, such as logistic or Gompertzian dynamics [5], whereas more recent approaches incorporate heterogeneous tumour compartments and resistance driven by mutation or pharmacological pressure [6, 7, 8]. Therefore, realistic chemotherapy models must capture resistance development, pharmacokinetics/pharmacodynamics (PK/PD), and clinical toxicity limits.

Fixed‐dose or single‐agent regimens often suppress drug‐sensitive cells but fail to control resistant subpopulations, frequently leading to tumour regrowth [9]. These limitations have motivated the development of adaptive dosing strategies that can account for tumour heterogeneity and maintain toxicity within allowable clinical bounds. Several control and optimisation techniques, including PID controllers [10, 11], internal model control [12], robust control [13], and multiobjective evolutionary algorithms [14, 15], have been investigated. Although these methods offer valuable insights, they often rely on precise model knowledge and may not fully represent the complex nonlinearities of tumour–drug interactions or the emergence of multidrug resistance.

Reinforcement learning (RL) has recently emerged as a promising framework for adaptive chemotherapy optimisation under uncertainty. RL enables the derivation of closed‐loop dosing strategies by interacting directly with a simulated physiological environment, without requiring an explicit analytical model of tumour dynamics. Among RL methods, Q‐learning is particularly suitable for chemotherapy because its discrete action structure aligns well with clinically approved dose levels. However, classical Q‐learning is prone to action‐value overestimation, which can destabilise learning in nonlinear PK/PD systems with delayed drug effects.

Double Q‐learning alleviates this limitation by decoupling action selection from value evaluation, resulting in more reliable and stable policy learning. Despite these advantages, its application to multidrug chemotherapy, particularly to triple‐agent regimens involving cisplatin, docetaxel and irinotecan, has not been systematically investigated. This gap is clinically important: although doublet regimens, such as cisplatin–docetaxel or cisplatin–irinotecan achieve objective response rates (ORRs) of 20%–35%, triple‐agent combinations have demonstrated improved ORRs of 30%–40% in NSCLC [16, 17], but optimal dosing strategies remain unclear due to toxicity constraints and heterogeneous resistance patterns. This gap highlights the need for computational methods capable of optimising multidrug dosing strategies for such regimens. Direct clinical translation is beyond the scope of this simulation‐based study.

This work develops a double Q‐learning–based controller to optimise a clinically motivated regimen combining cisplatin, docetaxel and irinotecan. The underlying PK/PD‐driven tumour model, adapted from ref. [18], is extended to incorporate eight resistance states and non‐cross‐resistance among the three drugs. A multiobjective reward structure is formulated to balance tumour suppression, toxicity minimisation and dose constraints. The proposed controller is trained to provide daily dosing recommendations tailored to simulated physiological dynamics. Robustness is thoroughly evaluated under ± 50% physiological parameter uncertainty and abrupt disturbance events mimicking mutation‐driven aggressiveness. The controller's performance is benchmarked against the IMC–FOPI controller in simulation of Pachauri et al. [19].

The major contributions of this study are summarised as follows:

- A refined PK/PD‐based three‐drug tumour model that incorporates noncross‐resistance dynamics, dose limits and clinical toxicity thresholds.
- Development of a double Q‐learning chemotherapy controller that mitigates overestimation bias and yields stable, adaptive multidrug scheduling policies.
- A multiobjective reward function designed to balance tumour suppression, toxicity regulation and dosage constraints in a clinically relevant manner.
- Comprehensive robustness analysis under up to ± 50% physiological parameter variations and abrupt tumour disturbances.
- Comparative analysis demonstrating improved performance over IMC–FOPI for nonlinear multidrug chemotherapy control.

In addition to biomedical modelling approaches, chemotherapy scheduling can be interpreted as a structured control problem belonging to several well‐studied classes of dynamical systems. First, pharmacokinetic absorption and elimination introduce inherent time delays between drug administration and therapeutic effect, making the system analogous to classical time‐delay processes commonly studied in industrial control. Second, tumour evolution under multiagent cytotoxic pressure exhibits strong nonlinearities, parameter uncertainty and time‐varying behaviour due to mutation‐driven resistance, which closely resembles uncertain nonlinear systems with drift dynamics. Third, clinical toxicity constraints impose strict bounds on admissible control inputs, whereas the simultaneous administration of multiple chemotherapeutic agents introduces coupling effects between control channels, resulting in a constrained multivariable control problem.

These structural similarities have motivated extensive research in advanced process control. Time‐delay compensation and prediction‐based structures, such as internal model control (IMC) and its cascade or dual‐loop variants, have been widely applied to delay‐dominated industrial systems due to their ability to explicitly handle transport lags and improve closed‐loop stability [12, 20]. In parallel, fractional‐order PID and PIDF‐based controllers have been shown to enhance robustness and memory‐dependent dynamic compensation in nonlinear systems with modelling uncertainty and disturbance sensitivity [11, 21, 22]. For highly nonlinear and uncertain systems, nonlinear backstepping approaches provide Lyapunov‐based stability guarantees under structured model assumptions, particularly in biochemical and reactor‐type systems [23]. Furthermore, optimisation‐driven tuning methods, including particle swarm optimisation and related evolutionary strategies, have been widely employed to improve controller performance in complex industrial processes with nonlinear and multivariable interactions [14, 15, 24].

Recent developments have further extended these ideas to more complex and realistic engineering applications, including decoupled IMC–PD structures for delay‐dominated chemical systems, stability‐constrained metaheuristic tuning of dual‐loop controllers and experimentally validated cascade control architectures for unstable and integrating processes. Such approaches demonstrate strong performance in terms of disturbance rejection, robustness to parameter variation and improved transient behaviour in industrial settings.

However, despite these advances, most of these control strategies fundamentally rely on either explicit system identification, structured parametric models or fixed controller architectures. In chemotherapy applications, these assumptions become restrictive due to continuously evolving tumour heterogeneity, emergence of drug resistance and interpatient variability, all of which induce nonstationary and partially unobservable system dynamics. As a result, classical model‐based controllers may experience reduced adaptability under distributional shifts in tumour evolution and pharmacological response.

In contrast, reinforcement learning (RL) provides a model‐free framework in which control policies are learnt directly through interaction with the environment, without requiring explicit knowledge of system equations. This is particularly advantageous in chemotherapy scheduling, where the underlying PK/PD system is only partially known and exhibits strong nonlinearities and time‐varying behaviour. Within this framework, Q‐learning aligns naturally with discretised clinical dosing strategies, whereas double Q‐learning further improves stability by reducing overestimation bias in action‐value estimation, thereby enhancing policy robustness in nonlinear and constrained environments.

The organisation of the paper is as follows: Section 2 presents a mathematical formulation of the three‐drug chemotherapy model. Section 3 describes the development of the double Q‐learning–based dosing controller. Section 4 reports simulation results and comparative analysis. Lastly, Section 5 provides a conclusion to the paper. The limitations and future research directions are discussed in Section 6.

## 2 Dynamic Model of Three‐Drug Cancer Chemotherapy

This section presents a mechanistic model describing the evolution of a heterogeneous tumour exposed to three chemotherapeutic agents. The formulation is adapted from the pharmacokinetics/pharmacodynamics (PK/PD) framework of ref. [18], which is widely used due to its ability to capture mutation‐driven resistance, drug–tumour interactions and multiagent dosing effects with tractable computational complexity. The in‐depth modelling supports the proper depiction of tumour development during multidrug chemotherapy with realistic pharmacological and biological hypotheses. The tumour population is divided into eight subpopulations according to their sensitivity or resistance to three chemotherapeutic agents. The fully sensitive compartment is denoted by S(t), whereas $R_{1}\left(t\right)$, $R_{2}\left(t\right)$ and $R_{3}\left(t\right)$ represent cells resistant to each single drug. Higher‐order resistance is modelled by $R_{12}\left(t\right)$, $R_{13}\left(t\right)$ and $R_{23}\left(t\right)$ and $R_{123}\left(t\right)$ captures complete resistance to all three drugs. Based on the above classification of drug‐response subpopulations, the total tumour mass R(t) can be expressed as the sum of all eight compartments:

(1) $$R\left(t\right)=S\left(t\right)+R_{1}\left(t\right)+R_{2}\left(t\right)+R_{3}\left(t\right)+R_{12}\left(t\right)+R_{13}\left(t\right)+R_{23}\left(t\right)+R_{123}\left(t\right)$$

The complete classification of resistance states is summarised in Table 1. This decomposition is employed throughout the model to characterise tumour dynamics under multidrug chemotherapy. In this work, the temporal evolution of the tumour cell population R(t) is described using an exponential growth model consistent with mathematical oncology. In the absence of treatment, each tumour subpopulation is assumed to proliferate at a uniform intrinsic rate ρ consistent with standard exponential tumour growth assumptions:

(2) $$\frac{dR\left(t\right)}{dt}=\rho R\left(t\right)$$

where ρ>0 represents the shared exponential growth constant across all subpopulations, each corresponding to a distinct resistance profile to the administered chemotherapeutic agents.

**TABLE 1:** Classification of cancer cell populations based on drug resistance.

Cell category	Symbol	Description
Sensitive to all three drugs	S(t)	Sensistive to drugs 1, 2, and 3
Cells resistant to a single drug	$R_{1}\left(t\right)$	Resistant to drug 1 only
	$R_{2}\left(t\right)$	Resistant to drug 2 only
	$R_{3}\left(t\right)$	Resistant to drug 3 only
Cells resistant to two drugs	$R_{12}\left(t\right)$	Resistant to drugs 1 and 2
	$R_{13}\left(t\right)$	Resistant to drugs 1 and 3
	$R_{23}\left(t\right)$	Resistant to drugs 2 and 3
Fully resistant population	$R_{123}\left(t\right)$	Resistant to all three drugs

Resistance is acquired through mutation with drug‐specific probabilities $\varphi_{i}$, and the model assumes no cross‐resistance between drugs, consistent with several cytotoxic classes. Let $C_{i}\left(t\right)$ denote the concentrations of i={1,2,3} chemotherapeutic agents, at the tumour site at a given time. Drug i kills only those cells that remain sensitive to it, and only when its concentration $C_{i}\left(t\right)$ exceeds the efficacy threshold $C_{\mathrm{thi}}$, represented via the Heaviside step function:

(3) $$H\left(C_{i}-C_{\mathrm{thi}}\right)=\left\{\begin{array}{cc} 1 & C_{i}>C_{\mathrm{thi}},\\ 0 & \text{otherwise}. \end{array}\right.$$

The tumour is decomposed into eight subpopulations based on their sensitivity or resistance to each of the three drugs. The fully sensitive compartment is denoted by S(t), representing cells that remain sensitive to all three drugs.

(4) $$\begin{array}{l} \frac{dS\left(t\right)}{dt}=\rho \left[\left(1-\varphi_{1}-\varphi_{2}-\varphi_{3}\right)S\left(t\right)\right]-k_{1}\left(C_{1}-C_{\mathrm{th1}}\right)H\left(C_{1}-C_{\mathrm{th1}}\right)S\left(t\right)-k_{2}\left(C_{2}-C_{\mathrm{th2}}\right)H\left(C_{2}-C_{\mathrm{th2}}\right)S\left(t\right)\\ \;-k_{3}\left(C_{3}-C_{\mathrm{th3}}\right)H\left(C_{3}-C_{\mathrm{th3}}\right)S\left(t\right) \end{array}$$

$k_{i}$ is only the fraction of population cells that are killed by effective chemotherapy. Sensitive cells possibility spontaneously develop resistance to drug i={1,2,3}.

Three additional subpopulations describe cells resistant to a single drug whereas remaining sensitive to the other two: $R_{1}\left(t\right)$ (resistant to Drug 1 only), $R_{2}\left(t\right)$ (resistant to Drug 2 only) and $R_{3}\left(t\right)$ (resistant to Drug 3 only).

(5) $$\begin{array}{l} \frac{dR_{1}\left(t\right)}{dt}=\rho \left[\left(1-\varphi_{2}-\varphi_{3}\right)\;R_{1}\left(t\right)+\varphi_{1}\;S\left(t\right)\right]\\ \;-\left[\;k_{2}\;\left(C_{2}-C_{\mathrm{th2}}\right)\;H\left(C_{2}-C_{\mathrm{th2}}\right)+k_{3}\;\left(C_{3}-C_{\mathrm{th3}}\right)\;H\left(C_{3}-C_{\mathrm{th3}}\right)\;\right]\;R_{1}\left(t\right) \end{array}$$

(6) $$\begin{array}{l} \frac{dR_{2}\left(t\right)}{dt}=\rho \left[\left(1-\varphi_{1}-\varphi_{3}\right)\;R_{2}\left(t\right)+\varphi_{2}\;S\left(t\right)\right]\\ \;-\left[\;k_{1}\;\left(C_{1}-C_{\mathrm{th1}}\right)\;H\left(C_{1}-C_{\mathrm{th1}}\right)+k_{3}\;\left(C_{3}-C_{\mathrm{th3}}\right)\;H\left(C_{3}-C_{\mathrm{th3}}\right)\;\right]\;R_{2}\left(t\right) \end{array}$$

(7) $$\begin{array}{l} \frac{dR_{3}\left(t\right)}{dt}=\rho \left[\left(1-\varphi_{1}-\varphi_{2}\right)\;R_{3}\left(t\right)+\varphi_{3}\;S\left(t\right)\right]\\ \;-\left[\;k_{1}\;\left(C_{1}-C_{\mathrm{th1}}\right)\;H\left(C_{1}-C_{\mathrm{th1}}\right)+k_{2}\;\left(C_{2}-C_{\mathrm{th2}}\right)\;H\left(C_{2}-C_{\mathrm{th2}}\right)\;\right]\;R_{3}\left(t\right) \end{array}$$

Higher‐order resistance states are captured through $R_{12}\left(t\right)$, $R_{13}\left(t\right)$ and $R_{23}\left(t\right)$, which describe resistance to two drugs whereas remaining sensitive to the third. Multidrug resistance arises from multiple mutations in tumour cells.

(8) $$\frac{dR_{12}\left(t\right)}{dt}=\rho \left[\left(1-\varphi_{3}\right)R_{12}\left(t\right)+\varphi_{2}R_{1}\left(t\right)+\varphi_{1}R_{2}\left(t\right)\right]-k_{3}\left(C_{3}-C_{\mathrm{th3}}\right)H\left(C_{3}-C_{\mathrm{th3}}\right)R_{12}\left(t\right)$$

(9) $$\frac{dR_{13}\left(t\right)}{dt}=\rho \left[\left(1-\varphi_{2}\right)R_{13}\left(t\right)+\varphi_{3}R_{1}\left(t\right)+\varphi_{1}R_{3}\left(t\right)\right]-k_{2}\left(C_{2}-C_{\mathrm{th2}}\right)H\left(C_{2}-C_{\mathrm{th2}}\right)R_{13}\left(t\right)$$

(10) $$\frac{dR_{23}\left(t\right)}{dt}=\rho \left[\left(1-\varphi_{1}\right)R_{23}\left(t\right)+\varphi_{2}R_{3}\left(t\right)+\varphi_{3}R_{2}\left(t\right)\right]-k_{1}\left(C_{1}-C_{\mathrm{th1}}\right)H\left(C_{1}-C_{\mathrm{th1}}\right)R_{23}\left(t\right)$$

A fully resistant population, denoted by $R_{123}\left(t\right)$, accounts for cells that are resistant to all three therapeutic agents.

(11) $$\frac{dR_{123}}{dt}=\rho R_{123}\left(t\right)+\rho \left[\varphi_{1}R_{23}\left(t\right)+\varphi_{2}R_{13}\left(t\right)+\varphi_{3}R_{12}\left(t\right)\right]$$

**FIGURE 1:**
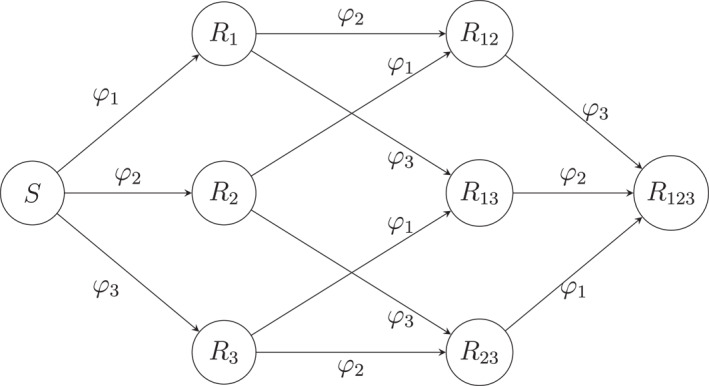
Mutation‐driven transitions among tumour resistance states.

The dynamics of the eight tumour subpopulations are given by Equations ((4), (5), (6), (7), (8), (9), (10), (11)). Figure 1 illustrates the mutation‐driven transitions among resistance states.

Apart from studying the tumour cell populations, this research also addresses drug toxicity and drug concentration. It is assumed that drug concentrations follow first‐order elimination kinetics. Hence, Equations ((12), (13), (14)) describe the behaviour of drug concentration over time:

(12) $$\frac{dC_{1}\left(t\right)}{dt}=u_{1}\left(t\right)-\lambda_{1}C_{1}\left(t\right),$$

(13) $$\frac{dC_{2}\left(t\right)}{dt}=u_{2}\left(t\right)-\lambda_{2}C_{2}\left(t\right),$$

(14) $$\frac{dC_{3}\left(t\right)}{dt}=u_{3}\left(t\right)-\lambda_{3}C_{3}\left(t\right),$$

where $u_{i}\left(t\right)$ is the administered dose and $\lambda_{i}$ denotes the elimination constant of drug i. This commonly used approximation captures the essential PK behaviour of cytotoxic agents for control design.

The relationship between drug concentration $C_{i}\left(t\right)$ and its cumulative toxicity $T_{i}\left(t\right)$ can be mathematically expressed according to Equations ((15), (16), (17)).

(15) $$\frac{dT_{1}\left(t\right)}{dt}=C_{1}\left(t\right)-\beta_{1}T_{1}\left(t\right),$$

(16) $$\frac{dT_{2}\left(t\right)}{dt}=C_{2}\left(t\right)-\beta_{2}T_{2}\left(t\right),$$

(17) $$\frac{dT_{3}\left(t\right)}{dt}=C_{3}\left(t\right)-\beta_{3}T_{3}\left(t\right),$$

where $\beta_{i}$ represents the metabolic clearance rate. Higher drug concentrations lead to increased toxicity and slower physiological recovery.

Equations ((1), (2), (3), (4), (5), (6), (7), (8), (9), (10), (11), (12), (13), (14), (15), (16), (17)) together define the transition dynamics used by the reinforcement‐learning controller.

## 3 Development of the Control Framework

### 3.1 Chemotherapy Scheduling as an RL Problem

The multidrug chemotherapy scheduling problem is formulated as a sequential decision‐making task in which an agent determines the optimal daily dosing vector for three cytotoxic drugs whilst accounting for nonlinear tumour‐drug interactions, cumulative toxicity and evolving resistance dynamics. The environmental dynamics follow the PK/PD and multicompartment tumour model defined in the previous section. The state vector integrates total tumour burden and cumulative toxicity levels associated with each chemotherapeutic drug:

(18) $$s\left(t\right)=\left[\;R\left(t\right),\;T_{1}\left(t\right),\;T_{2}\left(t\right),\;T_{3}\left(t\right)\;\right]$$

Each drug is administered at one of seven discrete dose levels motivated by clinically used dosing ranges for cytotoxic chemotherapy. The action space a(t) at time t is a three‐dimensional dosage vector:

(19) $$\begin{array}{c} a\left(t\right)=\left(u_{1}\left(t\right),\;u_{2}\left(t\right),\;u_{3}\left(t\right)\right),\;u_{i}\left(t\right)\in \left\{0,\;5,\;10,\;20,\;30,\;40,\;50\right\},\;i=1,2,3 \end{array}$$

A multiobjective reward function encourages tumour reduction whilst penalising toxicity above the clinical limit. Consequently, partial observability and nonlinear dynamics characterise the environment, agent learns an effective chemotherapy scheduling policy. The reward r(t) at each time step is defined as follows:

(20) $$r\left(t\right)=w_{1}\cdot \frac{R\left(t\right)-R^{\prime }\left(t\right)}{R\left(t\right)}-w_{2}\cdot \max\left(0,T_{1}\left(t\right)+T_{2}\left(t\right)+T_{3}\left(t\right)-T_{\max}\right),$$

R(t) denotes the number of tumour cells at time step t and $T_{i}\left(t\right)$ represents the toxicity level associated with the administered chemotherapy dose. Where $R^{\prime }\left(t\right)$ denotes the total tumour cell population after action a(t) was applied, $T_{\max}$ is the toxicity constraint threshold threshold and $w_{1}$ and $w_{2}$ are positive weighting coefficients reflecting the relative importance of tumour reduction versus toxicity control. This formulation is designed to incentivise actions that achieve larger proportional reductions in tumour mass whilst accounting for the cumulative toxicity of administered drugs. The max operator ensures that toxicity exceeding the predefined threshold is penalised within the reward function.

### 3.2 Classical Q‐Learning for Multi‐Drug Chemotherapy Optimisation

Q‐learning is a model‐free reinforcement learning (RL) method that enables an agent to learn optimal decision‐making strategies without requiring an explicit model of the underlying system dynamics. This property makes it suitable for biomedical applications, where physiological processes are complex and difficult to model analytically. Given this context, modelling chemotherapy dosing as a discrete‐action decision problem aligns closely with current medical standards, reduces computational complexity and enhances early‐stage research stability. The approach not only solves the problem of drug resistance but also considers the variability in patient response, presenting an adaptive approach for the treatment of cancer on an individualised level.

**FIGURE 2:**
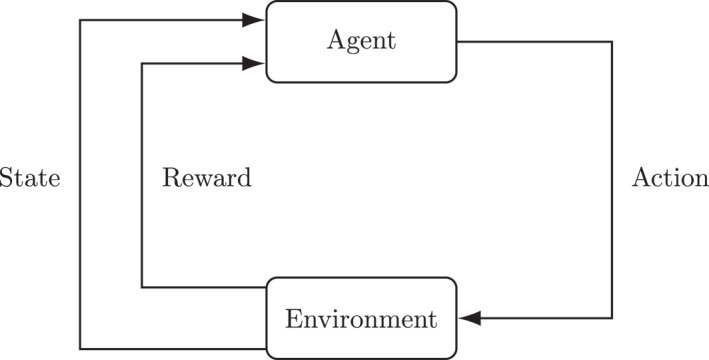
Interaction between the agent and the environment with state and reward signals.

As shown in Figure 2, the RL agent interacts with an emulated environment that simulates the behaviour of tumours and drugs. For each time step t, the agent in state s(t), following the selection of action a(t), receives the immediate reward r(t) from the environment. The agent learns an effective chemotherapy scheduling policy through repeated experience and trial‐and‐error interactions with the environment.

At each time step, the agent observes the current state and selects an action according to an ε‐greedy policy derived from the Q‐table. The Q‐value update rule helps the agents learn from the rewards earned, and the action selection policies get updated as time passes, which mixes the current Q‐value with the best‐discounted Q‐value for the next state and next action, weighted with α. Classical Q‐learning updates the action‐value estimate using:

(21) $$Q\left(s\left(t\right),a\left(t\right)\right)\leftarrow Q\left(s\left(t\right),a\left(t\right)\right)+\alpha \left[r\left(s\left(t\right),a\left(t\right)\right)+\gamma \max_{a^{\prime }}Q\left(s^{\prime }\left(t\right),a^{\prime }\left(t\right)\right)-Q\left(s\left(t\right),a\left(t\right)\right)\right]$$

The Q‐value Q(s(t),a(t)) approximates the expected total reward when action a(t) is taken in state s(t), as defined in Algorithm 1. The learning rate α determines how much new information affects the Q‐value updates. The discount factor γ balances the relative importance of future versus immediate rewards. Here, $s^{\prime }\left(t\right)$ is the next state and $a^{\prime }\left(t\right)$ refers to and next action that is chosen during the learning process.

ALGORITHM 1 **Classical Q‐Learning**

1

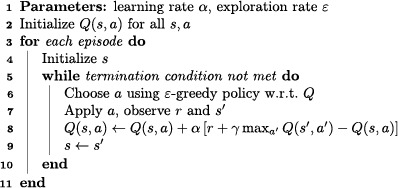

State transitions were governed by a custom environment function defined in Equations ((1), (2), (3), (4), (5), (6), (7), (8), (9), (10), (11), (12), (13), (14), (15), (16), (17)), which models the biological effects of chemotherapy using compartmental dynamics. The update incorporates:

- Evolution of the resistant and sensitive cell subpopulations $R^{\prime }\left(t\right)$,
- Cumulative toxicity: $T_{i}\left(t+1\right)$,
- The nonlinear effect of drugs according to present dosing and resistance phenotype.
- Constraints were imposed to maintain the administered drug dose within predefined upper bounds consistent with clinical dosing ranges:

(22) $$u_{i}\leq u_{\text{max}},\;\forall i\in \left\{1,2,3\right\}.$$

where $u_{i}\left(t\right)$ is the dose of drug i at time step t and $u_{\text{max}}=50$ is derived from established clinical guidelines.

This formulation explicitly defines both objectives and constraints, providing a clear framework for reinforcement learning‐based optimisation of multidrug chemotherapy regimens such that the learnt policy aims to maximise tumour reduction whilst maintaining toxicity within acceptable clinical limits. However, the max operator causes systematic overestimation of action values, which can destabilise learning in nonlinear PK/PD environments with delayed drug effects. As a result, random estimation noise leads to optimistic value updates and unstable learning, particularly in nonlinear control settings.

### 3.3 Double Q‐Learning for Multi‐Drug Chemotherapy Optimisation

Double Q‐learning addresses this issue by maintaining two independent estimators, $Q_{A}$ and $Q_{B}$, and decoupling the action‐selection and value‐evaluation roles. This leads to more accurate Bellman targets and significantly more stable policy learning, an essential requirement for toxicity‐constrained chemotherapy scheduling. In chemotherapy scheduling, unstable policies may generate abrupt dosing changes that are not practically desirable or may violate prescribed dosing constraints.

For action selection during interaction, the agent uses an ε‐greedy policy derived from the combined estimate:

(23) $$a\left(t\right)=\left\{\begin{array}{cc} \text{random}\;\text{action}, & \text{with}\;\text{probability}\;\varepsilon ,\\ \arg\max_{a}\left(Q_{A}\left(s\left(t\right),a\right)+Q_{B}\left(s\left(t\right),a\right)\right), & \text{otherwise}. \end{array}\right.$$

At each update step, one of the two estimators is randomly selected (with probability 0.5) to perform the update, whereas the other is used for action evaluation. The update rule is as follows:

(24) $$\begin{array}{cc} \text{if}\;\omega <0.5:\; & Q_{A}\left(s\left(t\right),a\left(t\right)\right)\leftarrow Q_{A}\left(s\left(t\right),a\left(t\right)\right)+\alpha \left[r\left(t\right)+\gamma \;Q_{B}\;\left(s^{\prime }\left(t\right),a^{*}\left(t\right)\right)-Q_{A}\left(s\left(t\right),a\left(t\right)\right)\right]\\ \text{else:}\; & Q_{B}\left(s\left(t\right),a\left(t\right)\right)\leftarrow Q_{B}\left(s\left(t\right),a\left(t\right)\right)+\alpha \left[r\left(t\right)+\gamma \;Q_{A}\;\left(s^{\prime }\left(t\right),a^{*}\left(t\right)\right)-Q_{B}\left(s\left(t\right),a\left(t\right)\right)\right] \end{array}$$

In double Q‐learning, the action used for the target is selected by the estimator chosen through the random switching variable ω:

(25) $$a^{*}=\left\{\begin{array}{cc} \arg\max_{a^{\prime }}Q_{A}\left(s^{\prime },a^{\prime }\right), & \omega <0.5,\\ \arg\max_{a^{\prime }}Q_{B}\left(s^{\prime },a^{\prime }\right), & \omega >0.5. \end{array}\right.$$

When $Q_{A}$ is updated (i.e., ω<0.5), the target is evaluated using $Q_{B}\left(s^{\prime },a^{*}\right)$; when $Q_{B}$ is updated, the target uses $Q_{A}\left(s^{\prime },a^{*}\right)$. This preserves the separation between action selection and action evaluation, eliminating the overestimation bias of standard Q‐learning.

In double Q‐learning Algorithm 2, $Q_{A}\left(s\left(t\right),a\left(t\right)\right)$ and $Q_{B}\left(s\left(t\right),a\left(t\right)\right)$ denote the Q‐values in the two independent Q‐tables. Finally, the next state $s^{\prime }\left(t\right)$ and next action $a^{\prime }\left(t\right)$ are chosen during the learning process.

ALGORITHM 2 **: Double Q‐Learning**

1

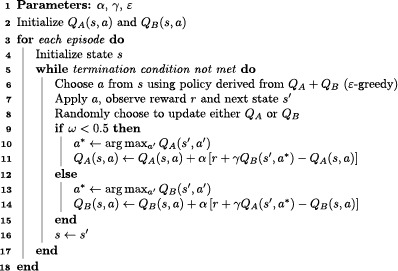

Through this alternating‐update structure, double Q‐learning generates unbiased value estimates and avoids the optimistic bias inherent in classical Q‐learning. Under the chemotherapy reward structure (Equation 20), the controller learns clinically feasible daily dosing strategies that minimise tumour burden whilst respecting toxicity limits. This formulation is identical to the standard double Q‐learning update described by Hasselt [25] and reduces overestimation bias in action‐value estimation. The performance of the proposed double Q‐learning framework is compared against the IMC–FOPI controller in ref. [19].

## 4 Simulation Results

### 4.1 Initial Values and Constraints

In this work, the model is trained and validated under the assumption that all patients have advanced‐stage lung cancer, with homogeneous growth and mutation rates. The multidrug chemotherapy scheduling problem is formulated as a reinforcement learning environment, in which a simulated biological system is interacted with by a double Q‐learning agent to learn optimal drug dosing policies over a maximum treatment horizon of 84 days with a daily time step (Δt=1). The simulation captures tumour growth, drug resistance dynamics and toxicity accumulation across multiple cell populations.

The initial values for the three‐drug model parameters were determined by Tse et al. [18] through memetic algorithm‐based chemotherapy model optimisation. All initial values are listed in Table 2.

**TABLE 2:** The three‐drug model parameters and their initial values.

Symbol	Description	Value	Unit
$\beta_{1}$	Constant related to the metabolism rate of drug 1	0.4	$day^{-1}$
$\beta_{2}$	Constant related to the metabolism rate of drug 2	0.5	$day^{-1}$
$\beta_{3}$	Constant related to the metabolism rate of drug 3	0.45	$day^{-1}$
$\varphi_{1}$	Mutation rate from sensitive subpopulation to resistant to drug 1	0.008	$day^{-1}$
$\varphi_{2}$	Mutation rate from sensitive subpopulation to resistant to drug 2	0.01	$day^{-1}$
$\varphi_{3}$	Mutation rate from sensitive subpopulation to resistant to drug 3	0.014	$day^{-1}$
$\lambda_{1}$	Decay constant of drug 1	0.32	$day^{-1}$
$\lambda_{2}$	Decay constant of drug 2	0.27	$day^{-1}$
$\lambda_{3}$	Decay constant of drug 3	0.25	$day^{-1}$
ρ	Constant related to the growth rate	0.0099	—
S(0)	Total sensitive population	$4.60517\times 10^{11}$	cells
$k_{1}$	Fraction of cells killed by drug 1 per unit time and drug concentration	0.0084	$day^{-1}D^{-1}$
$k_{2}$	Fraction of cells killed by drug 2 per unit time and drug concentration	0.0074	$day^{-1}D^{-1}$
$k_{3}$	Fraction of cells killed by drug 3 per unit time and drug concentration	0.0092	$day^{-1}D^{-1}$
$C_{\mathrm{th1}}$	Threshold concentration of drug 1 for effectiveness	10	D
$C_{\mathrm{th2}}$	Threshold concentration of drug 2 for effectiveness	10	D
$C_{\mathrm{th3}}$	Threshold concentration of drug 3 for effectiveness	10	D

To ensure patient safety during chemotherapy scheduling, strict dose–safety constraints were incorporated into the controller. For each treatment step, the allowable infusion rate for cisplatin, docetaxel and irinotecan was limited to a maximum of 50 ${mg/m}^{2}$, which is below their standard single‐agent doses (cisplatin: 50–75 ${mg/m}^{2}$, docetaxel: 75 ${mg/m}^{2}$ and irinotecan: 125 ${mg/m}^{2}$ per cycle) as recommended in FDA/NCCN guidelines [26, 27, 28, 29, 30]. A real‐time toxicity index was continuously monitored, and doses were automatically reduced or withheld if toxicity approached predefined safety thresholds. These mechanisms are designed to keep controller‐generated drug schedules within predefined dosing constraints whilst maintaining tumour suppression performance in the simulation. Although direct patient testing has not yet been performed, the simulated results provide an initial assessment of the model's potential efficacy and safety in a controlled setting. A direct correlation exists between drug toxicity and cancerous cell reduction. However, this cytotoxic effect adversely impacts patients' physiological conditions. Consequently, assessing drug toxicity within tolerable thresholds is equally critical.

(26) $$\left\{\begin{array}{c} 10<C_{i}\left(t\right)\leq 50\\ T_{i}\left(t\right)\leq 100\\ u_{i}\left(t\right)\leq 50 \end{array}\right.\;\text{for}\;i\in \left\{1,2,3\right\}$$

Cancer cells multiply exponentially, and the volume of the malignant mass is measured volumetrically in the laboratory. In this work, the malignant cell mass will be presented in units in the form of $4.60517\times 10^{11}$ cells. Here, the constraint rule is that:

(27) $$R\left(t\right)\leq S\left(0\right)\;also\;\lim_{t\to \infty }R\left(t\right)=0$$

Parameters of Q‐learning and double Q‐learning are in Table 3. The learning rate (α=0.01) and discount factor (γ=0.99) have been widely adopted in prior studies for discrete‐action Q‐learning and double Q‐learning [25, 31].

**TABLE 3:** Double Q‐learning and Q‐learning algorithm parameters.

Symbol	Description	Value
α	Learning rate	0.01
γ	Discount factor	0.99
ϵ	Exploration probability	1.0–0.01
—	Number of training episodes	5000
—	Evaluation episodes	5000
ω	Random number	0–1
$w_{1}$	Reward weighting coefficient	50
$w_{2}$	Reward weighting coefficient	0.5

### 4.2 Results

The observed differences in tumour reduction among IMC–FOPI, Q‐learning and double Q‐learning can be interpreted through the interaction between policy stability and treatment aggressiveness in a nonlinear PK/PD environment. Although all controllers initially induce rapid tumour decline due to high early dosing, their long‐term behaviour diverges as resistance dynamics and toxicity constraints become dominant. IMC–FOPI relies on a fixed control law and cannot adapt to evolving tumour heterogeneity, leading to suboptimal balancing between drug exposure and resistance emergence. In contrast, Q‐learning adapts dosing based on learnt action values but suffers from overestimation bias, which intermittently favours overly aggressive dosing decisions. This produces unstable suppression dynamics and allows residual resistant populations to persist. Double Q‐learning mitigates this effect by decoupling action selection from value estimation, resulting in more conservative and consistent dosing trajectories. Consequently, the controller maintains sustained suppression pressure without inducing destabilising oscillations in tumour burden, leading to substantially improved tumour control over the treatment horizon.

**FIGURE 3:**
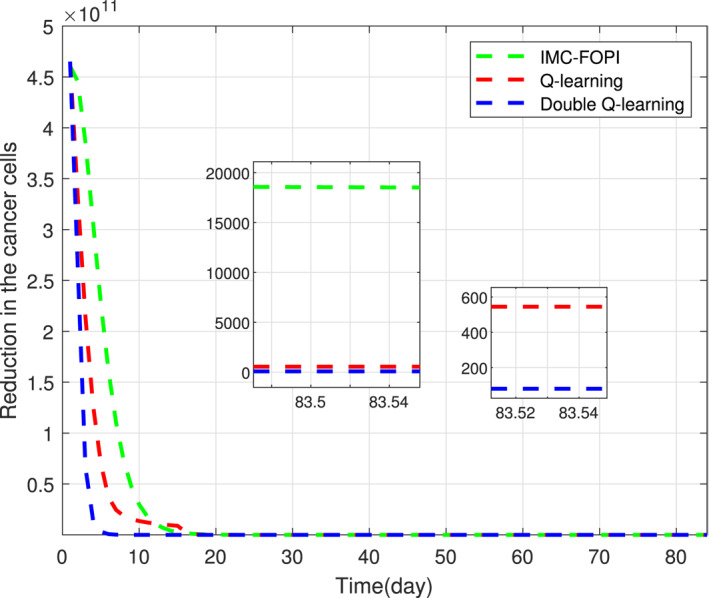
Reduction in number of cancer cells (*R*).

Figure 3 illustrates the temporal evolution of the total cancer cell population R(t) under IMC–FOPI, Q‐learning and double Q‐learning controllers. All methods achieve an initial rapid reduction in tumour burden during the early treatment phase (approximately the first 15 days). However, their long‐term suppression outcomes differ significantly. IMC–FOPI exhibits the weakest performance, with noticeable tumour persistence and partial regrowth tendencies during the mid‐to‐late treatment stages. Q‐learning achieves improved suppression compared to IMC–FOPI but still leaves a measurable residual tumour population due to suboptimal action‐value estimation. In contrast, double Q‐learning achieves the lowest residual tumour burden, reducing the population to 80 cells (Table 4), corresponding to $1.7372\times 10^{-8}\%$ of the initial value and an overall reduction of approximately 99.999998%, demonstrating the highest control efficiency among the compared strategies.

**TABLE 4:** Performance for controllers in case 1.

Controller	Percent	Cancer cells	Toxicity
IMC‐FOPI	0%	17,583	202.03
	±10%	22,677	202.03
	±20%	27,300	202.03
	±50%	33,406	202.03
Q‐learning	0%	545	278.13
	±10%	644.21	278.13
	±20%	780.33	278.13
	±50%	1971.26	278.13
Double Q‐learning	0%	80	222.57
	±10%	80.60	222.57
	±20%	81.91	222.57
	±50%	83.64	222.57

The superior performance of double Q‐learning can be attributed to reduced overestimation bias in action‐value estimation, resulting in more stable policy updates. This improved stability leads to more consistent dose selection over the treatment horizon, preventing the intermittent overestimation‐driven escalation patterns observed in classical Q‐learning. As a consequence, double Q‐learning avoids overly aggressive dosing strategies that may temporarily enhance tumour suppression but increase toxicity and destabilise long‐term treatment dynamics.

**FIGURE 4:**
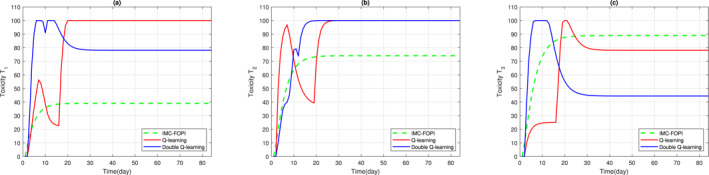
Toxicity variations for all designed controllers: (a) $T_{1}$, (b) $T_{2}$ and (c) $T_{3}$.

Figure 4a–c illustrates the temporal evolution of toxicity levels for drugs 1, 2 and 3 under IMC–FOPI, Q‐learning and double Q‐learning controllers. In all cases, toxicity increases during the early treatment phase due to intensified dosing required for tumour reduction, followed by convergence towards a steady‐state regime governed by system constraints. Drug‐specific variations arise from pharmacodynamic and decay‐rate differences, where drug 2 exhibits the highest toxicity peak (100) due to stronger cytotoxic interaction strength, whereas drug 3 exhibits the lowest toxicity level (44.4) consistent with its lower effective killing rate. Across control strategies, IMC–FOPI produces smoother but less adaptive toxicity regulation, Q‐learning induces more fluctuating trajectories due to instability in learnt value estimates and double Q‐learning achieves more consistent toxicity profiles through improved policy stability. These bounded toxicity dynamics indicate that all controllers implicitly satisfy the imposed safety constraints; however, the degree of efficiency in maintaining the toxicity–efficacy trade‐off differs significantly. In particular, double Q‐learning achieves a more balanced regulation by aligning long‐term reward maximisation with toxicity penalties, resulting in faster convergence to a stable operating regime compared to the other methods.

**FIGURE 5:**
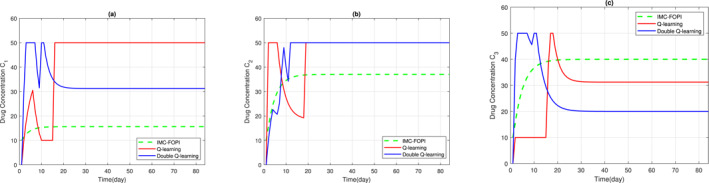
Drug concentrations variations: (a) $C_{1}$, (b) $C_{2}$ and (c) $C_{3}$.

**FIGURE 6:**
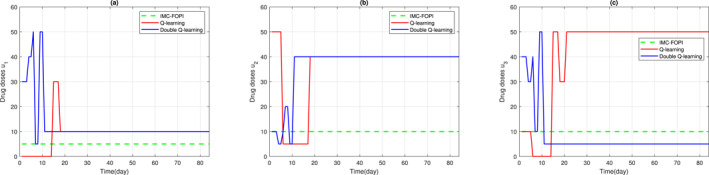
Individual drug doses: (a) $u_{1}$, (b) $u_{2}$ and (c) $u_{3}$.

Figure 5 presents the concentration of the drug at the tumour site, resulting from the scheduling strategies shown in Figure 6. During the initial treatment stages, the drug dosage gradually increases to its maximum value and then stabilises at the minimum level for all drugs in Figure 6. In all controllers, the concentration of the drug follows the same trends as in the dosage scheduling. The concentrations reach the corresponding pre‐established desired maximum values. Most importantly, these peak concentrations are below the maximal tolerable limits established.

**FIGURE 7:**
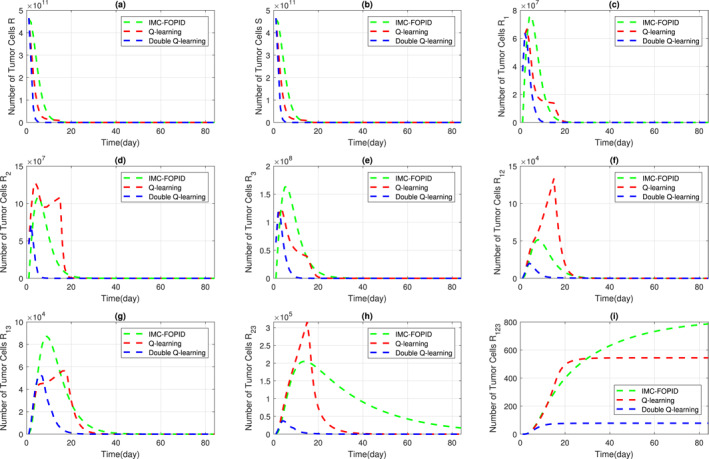
The subpopulation of cancer cells: (a) R, (b) S, (c) $R_{1}$ (d) $R_{2}$, (e) $R_{3}$, (f) $R_{12}$, (g)$R_{13}$, (h) $R_{23}$ and (i) $R_{123}$.

The total cancer cell population S(0) decreases and reaches its minimum level across all designed controllers, with double Q‐learning achieving a lower residual tumour burden compared to Q‐learning. This improvement is primarily attributed to the more stable and consistent dosing policy generated by double Q‐learning, which sustains multidrug pressure over the treatment horizon and reduces opportunities for resistant clone survival. In contrast, Q‐learning exhibits less stable action‐value estimation, leading to intermittent variations in dosing intensity that allow partial regrowth of resistant subpopulations. The detailed evolution of cancer cell subpopulations under double Q‐learning is illustrated in Figure 7a–i, where only 80 cells remain viable at the terminal stage, all of which exhibit resistance to all three drugs, indicating a strong selection pressure towards fully multiresistant phenotypes. Furthermore, as shown in Table 1, the administration of drug $u_{3}$ effectively suppresses subpopulation $R_{12}$, consistent with its sensitivity profile, since these cells are resistant only to $u_{1}$ and $u_{2}$, highlighting the role of differential drug sensitivity in shaping the final resistant landscape.

Clinical studies in patients with advanced or recurrent non‐small cell lung cancer (NSCLC) typically report progression‐free survival (PFS) values of approximately 4–7 months, depending on the therapeutic regimen and patient characteristics [16, 32]. In the proposed simulation framework, a surrogate PFS metric was defined as the time during which tumour burden remained below a predefined threshold over the 211‐day simulation horizon. Under the optimised chemotherapy scheduling learnt by double Q‐learning, tumour burden remained below this threshold for the entire simulation horizon, indicating sustained suppression within the simulated environment. This surrogate PFS should not be interpreted as clinical PFS equivalence as the model does not incorporate patient‐specific variability, toxicity grading systems or treatment cycle constraints. These results suggest that the proposed control strategy may provide an extended tumour suppression horizon under idealised conditions. The observed sustained suppression represents a full‐horizon maintenance of tumour control within the simulated environment. Compared with clinically reported NSCLC progression timelines (typically several months), the simulation indicates delayed progression under the idealised assumptions of the PK/PD model. This difference arises from simplified tumour dynamics, absence of interpatient variability and perfect state observability. Accordingly, the reported outcome should be interpreted as an upper‐bound performance of the proposed adaptive dosing policy under controlled simulation conditions rather than a clinically predictive estimate.

The improved performance of double Q‐learning is primarily associated with more stable action‐value estimation, which reduces variability in policy updates and results in more consistent dosing decisions. This improves the balance between tumour suppression and toxicity regulation compared with classical Q‐learning, which exhibits less stable policy behaviour due to overestimation effects in value estimation.

### 4.3 Robustness Analysis

Cancer progression and physiological responses exhibit considerable interpatient variability and evolve dynamically during treatment. Classical controller designs rely on fixed parameter assumptions, which may not reflect these clinical uncertainties. To evaluate closed‐loop performance under uncertainty, the robustness of both Q‐learning and double Q‐learning controllers is investigated through two scenarios: 4.3.1 stochastic perturbations applied to the tumour growth parameter R, representing sensor noise and modelling errors; and 4.3.2 abrupt shifts in R, simulating rapid tumour aggressiveness changes due to treatment resistance or unforeseen biological events.

#### 4.3.1 Robustness Under Parametric Uncertainty

To evaluate closed‐loop robustness, the tumour growth parameter R was perturbed by ±10%, ±20% and ±50% relative to its nominal value. Table 4 summarises the resulting cancer cell counts and toxicity levels for each controller. The IMC–FOPI controller showed high sensitivity to parameter variations, exhibiting more than a ≤90% increase in tumour burden under ±50% uncertainty. Q‐learning improved robustness, though tumour reduction remained sensitive to parameter changes, particularly under high perturbation. Double Q‐learning maintained tumour suppression with minimal deviation from the nominal case, showing only a ≤4.6% change even under ±50% parameter uncertainty, whilst keeping toxicity within clinically acceptable limits. Maintaining robust performance under large variations demonstrates reduced sensitivity to parametric variability within the simulated model, supports future methodological investigation.

Overall, the results show that all controllers are affected by parameter uncertainty, but learning‐based strategies demonstrate more stable behaviour than the model‐based baseline. Double Q‐learning exhibits the smallest deviation across uncertainty levels, suggesting better adaptability to physiological variability.

#### 4.3.2 Robustness to Abrupt Disturbances

**FIGURE 8:**
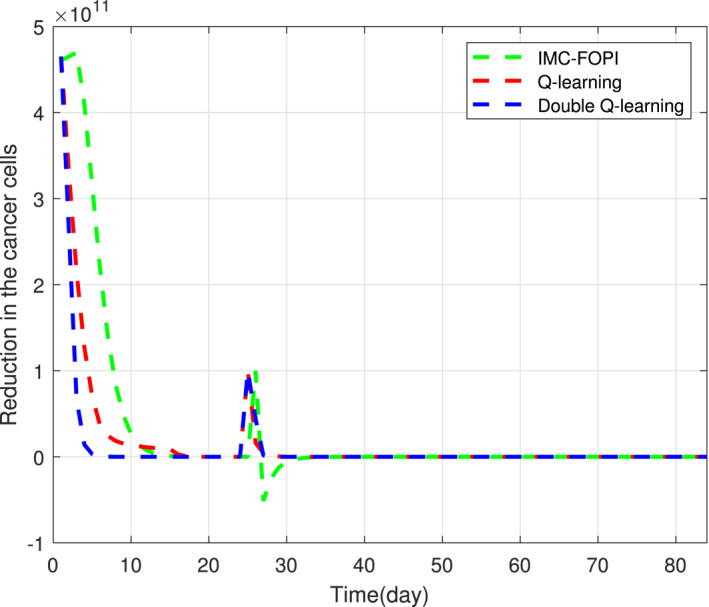
Reduction in the number of cancer cells (*R*) with disturbance.

In chemotherapy, the therapeutic process is often subject to disturbances such as genetic mutations or nonlinear responses. To further investigate the disturbance rejection capability, an abrupt increase in the tumour population ($D_{\mathrm{dis}}=10^{11}$ cells) was introduced at t=25 days, mimicking a sudden escalation in tumour aggressiveness due to treatment resistance. The amplitude of the injected perturbation is chosen to be sufficiently large such that the system performance experiences an appreciable degradation. The resulting tumour dynamics are shown in Figure 8 and Table 5. The IMC–FOPI controller exhibits a pronounced transient surge in tumour burden and shows a slow recovery, requiring nearly 10 days to return to its predisturbance trajectory, indicating limited robustness to aggressive tumour progression. Q‐learning recovers more rapidly than the baseline controller, although a short‐term degradation in performance is still observed immediately after the disturbance. Double Q‐learning demonstrates the most stable response, exhibiting only a small transient deviation and restoring tumour suppression in less than 2 days, without any oscillatory or unstable behaviour. Notably, no oscillatory response or loss of stability occurs, confirming that the learnt policy effectively adapts to the perturbed physiological state. These results highlight that double Q‐learning performs better in suppressing disturbance‐induced tumour expansion and maintaining stable system behaviour within the simulation framework.

**TABLE 5:** Performance for controllers in case 2.

Controller	Cancer cells	Toxicity
IMC‐FOPI	23,122.4	202.03
Q‐learning	551	278.13
Double Q‐learning	88.4	222.57

**FIGURE 9:**
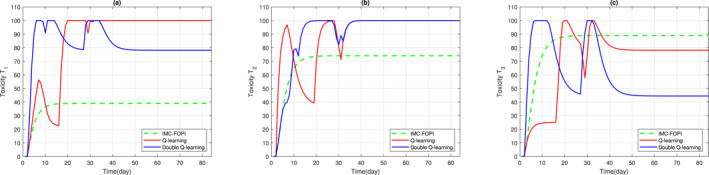
Toxicity variations for all designed controllers: (a) $T_{1}$, (b) $T_{2}$ and (c) $T_{3}$ with disturbance.

**FIGURE 10:**
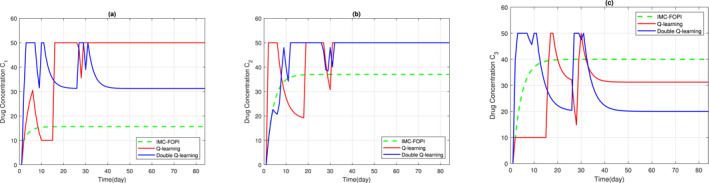
Drug concentrations variations: (a) $C_{1}$, (b) $C_{2}$ and (c) $C_{3}$ with disturbance.

**FIGURE 11:**
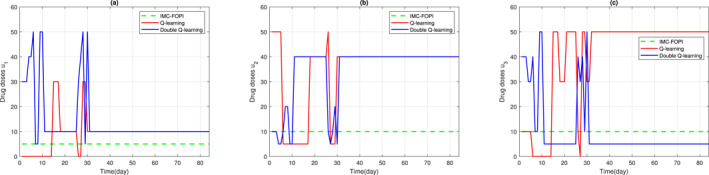
Individual drug doses: (a) $u_{1}$, (b) $u_{2}$ and (c) $u_{3}$ with disturbance.

Since the PID controller showed weak performance in disturbance rejection following the imposed disturbance at day 25, its toxicity, concentration and drug doses are not of significant importance. In contrast, the double Q‐learning controller demonstrated a substantially superior performance compared to the conventional Q‐learning algorithm as evident from the concentration, dose and toxicity profiles shown in Figures 9, 10, 11. The double Q‐learning controller maintains toxicity within a narrow and safe range throughout the entire therapy duration. This behaviour reflects a more cautious and physiologically compatible treatment policy, demonstrating that intelligent dose scheduling can successfully mitigate perturbations whilst maintaining efficacy and minimising overall toxicity.

**FIGURE 12:**
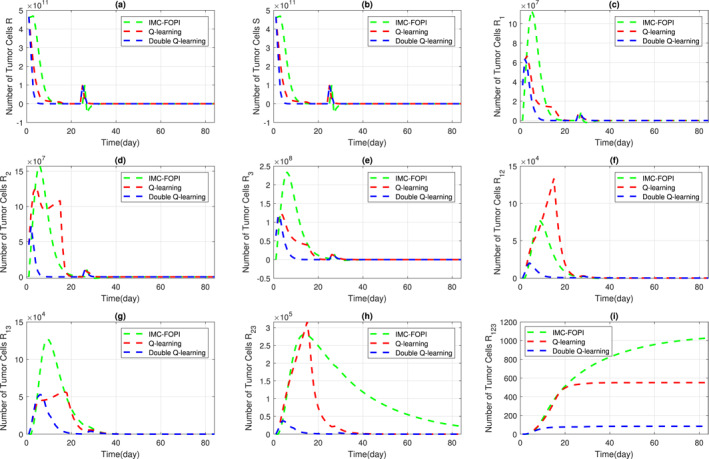
The subpopulation of cancer cells: (a) R, (b) S, (c) $R_{1}$ (d) $R_{2}$, (e) $R_{3}$, (f) $R_{12}$, (g) $R_{13}$, (h) $R_{23}$ and (i) $R_{123}$ with disturbance.

As Figure 12 shows, after the perturbation is injected, the IMC–FOPI controller is unable to effectively compensate for sudden tumour progression or biological variations. This behaviour suggests that IMC–FOPI lacks the robustness required for reliable clinical application in adaptive chemotherapy. Double Q‐learning leads to more stable and reliable learning outcomes in the multidrug scheduling setting. Although a transient increase occurs immediately after the disturbance, the controller rapidly compensates for the induced deviation and restores a stable trajectory within approximately 2 days.

The robustness observed under parametric uncertainty suggests that the learnt policy operates in a locally smooth region of the state‐action space, where small perturbations in tumour growth dynamics do not significantly alter the optimal action selection. This property is particularly important in biomedical applications, where interpatient variability introduces continuous shifts in system dynamics. These results indicate that learning‐based controllers adapt more effectively to sudden changes in tumour dynamics. Double Q‐learning demonstrates the strongest disturbance‐rejection capability, maintaining stability without inducing oscillatory behaviour.

## 5 Conclusion

This study introduced a double Q‐learning–based discrete‐action controller that uses dosing levels consistent with predefined clinical dosing ranges. The method was developed to optimise multidrug chemotherapy schedules, aiming for substantial tumour reduction whilst keeping toxicity within tolerable limits. The double Q‐learning controller, compared to other controllers, achieved tumour reduction exceeding (>99.9999%) within the simulation environment under nominal conditions. Validation of the reliability of the suggested method under large physiological uncertainties and external disturbances was provided by robustness analyses. The comparison of the dosages, concentration profiles and cancer cell dynamics was indicated by the simulations. Quantitatively, the proposed double Q‐learning controller achieved a tumour reduction exceeding 99.9999% under nominal conditions. Under ±50% parameter uncertainty, performance degradation remained below 4.6%, whereas Q‐learning and IMC–FOPI exhibited significantly higher sensitivity and instability. Furthermore, under abrupt disturbance injection, the proposed controller restored stable tumour suppression within approximately 2 days compared to approximately 10 days for IMC–FOPI and slower recovery for classical Q‐learning. These results demonstrate improved robustness, faster recovery and enhanced long‐horizon stability of the proposed method. Overall, this work demonstrates the potential of reinforcement learning for adaptive chemotherapy scheduling in a simulation‐based environment. The results should be interpreted as a methodological proof‐of‐concept rather than a clinically validated treatment framework.

## 6 Limitations and Future Work

This study has several limitations that should be explicitly acknowledged. First, the proposed controller is evaluated exclusively in a simulated PK/PD environment, and its performance is therefore inherently dependent on the accuracy and structural assumptions of the underlying mathematical model. No in vivo experiments or clinical or retrospective patient data have been used, and no claims are made regarding clinical efficacy or patient‐specific treatment outcomes. Second, the tumour model assumes homogeneous growth and mutation rates across all subpopulations, which does not capture the interpatient and intratumour heterogeneity typically observed in advanced cancer. In particular, variability in clonal evolution, spatial tumour structure and immune interactions are not represented. In addition, clinically relevant complexities, such as toxicity grading systems (e.g., CTCAE‐based classification), treatment cycle scheduling, dose delays and patient adherence, are not explicitly incorporated into the model. Third, pharmacokinetic and pharmacodynamic variability is only partially represented, and the controller is trained under a fixed distribution of initial conditions and parameter values. As a result, the learnt policy may be sensitive to deviations outside the training distribution, limiting its generalisability to broader and more diverse patient populations. Finally, the study relies on a single PK/PD model structure, and alternative tumour growth and resistance models may lead to different optimal control policies and potentially different qualitative system behaviour. Therefore, the results should be interpreted strictly as a proof‐of‐concept demonstration of reinforcement learning‐based chemotherapy scheduling in a controlled simulation environment. Future work will focus on validating the proposed framework using retrospective clinical datasets, incorporating patient‐specific pharmacokinetic variability and evaluating robustness under clinically realistic uncertainty distributions. Additional directions include domain randomisation during training, uncertainty‐aware reinforcement learning (e.g., ensemble or Bayesian Q‐learning variants) and off‐policy evaluation methods to improve reliability under distribution shift. Moreover, extending the framework to multiple PK/PD model classes and incorporating clinically structured toxicity grading systems will be essential steps towards improving translational relevance.

## Author Contributions

**Behnoush Alizade:** conceptualisation, data curation, formal analysis, investigation, methodology, resources, software, validation, visualisation, writing – original draft. **Ahmad Hajipour:** conceptualisation, funding acquisition, investigation, methodology, project administration, supervision, validation, writing – review and editing.

## Funding

The authors have nothing to report.

## Conflicts of Interest

The authors declare no conflicts of interest.

## Data Availability

The data that support the findings of this study are available from the corresponding author upon reasonable request.
